# Combating Prenatal Depression with Diet: A Literature Review on the Association Between Mediterranean or Mediterranean-like Diet Adherence and the Incidence of Depression in Pregnant Women

**DOI:** 10.3390/nu17071276

**Published:** 2025-04-06

**Authors:** Anna C. Malik, Sarah S. Comstock

**Affiliations:** Department of Food Science & Human Nutrition, Michigan State University, East Lansing, MI 48824, USA; malikann@msu.edu

**Keywords:** Mediterranean diet, depression, pregnancy

## Abstract

**Background/Objectives**: The purpose of this review is to determine if high adherence to the Mediterranean Diet or Mediterranean-like Diet is associated with a decreased incidence of depression or depressive symptoms during pregnancy compared to low or no adherence. **Methods**: PubMed was used to search for articles. Inclusion criteria consisted of primary research articles from the past 5 years, participants that were pregnant and ages 18–50 years old, the intervention a Mediterranean or Mediterranean-like Diet, and the outcome prenatal depression or prenatal depressive symptoms. **Results**: Nine primary research studies including five cross-sectional, two longitudinal, one cohort study, and one randomized controlled trial were included in this review. Six of the nine studies reported significant associations between higher Mediterranean or Mediterranean-like Diet adherence and lower incidence of depression or depressive symptoms. Studies with larger sample sizes were more likely to have significant results. **Conclusions**: Other recent evidence suggests that high adherence to the Mediterranean or Mediterranean-like Diet may decrease the incidence of depression or depressive symptoms in pregnant women. However, there were mixed results reported in the studies included in this review. Thus, conducting further studies of adequate sample sizes, with a unified definition of the Mediterranean Diet in more diverse populations is imperative to further investigate this association.

## 1. Introduction

Depression is a mood disorder that results in the feeling of hopelessness and sadness that can affect how one thinks and acts [1]. This common condition affects more women than men, and onset is common in women during their reproductive years [2]. According to a 2023 national survey, it is estimated that around 29% of adults have had a depression diagnosis at some point in their lifetime and around 18% of adults are currently experiencing depression [1]. Many are familiar with the term “post-partum depression,” which is the onset of depression after giving birth [3]. However, 7% to 9% of women in the United States experience depression during pregnancy, and rates may be higher worldwide [2]. Women experiencing depression during pregnancy are often less inclined to take care of themselves during this time and are more likely to participate in unwanted behaviors such as drinking, smoking, and poor nutritional intake [4]. These behaviors can lead to poor outcomes for not just the mother but also the unborn child, with increased risk of low birth weight and development issues [4]. Additionally, when a woman experiences depression during pregnancy, she has a higher risk of developing post-partum depression and having bonding or attachment issues with her child [2]. Therefore, treatment of depression during pregnancy is essential for the mother and child’s health.

There are discrepancies in how to treat depression during pregnancy, due to the concern of antidepressants affecting the unborn child [5]. However, other natural strategies to help treat depression may include support groups, exercise, and overall diet [5]. A 2022 review conducted by Selvaraj et al., discusses how the diet alters different physiological mechanisms including inflammation, oxidative stress, neuroplasticity, mitochondrial function, and the gut microbiome [6]. These mechanisms are also associated with neurological function, which may be the link between the association of dietary intake and the likelihood of developing depression [6]. Thus, the 2022 review investigated the association between diet and depression in adolescents and adults in observational studies and found that following a healthy diet, which incorporates vegetables and fruits, and exempts a pro-inflammatory diet including highly processed foods, refined carbohydrates, saturated fats, and high meat intake, may lower the risk of developing depressive symptoms or clinical depression [6]. The authors concluded that more non-observational studies should be conducted on the association between diet and depression due to the reported potential connection between the two in observational studies [6]. A diet that closely resembles this description mentioned above is the Mediterranean Diet.

The Mediterranean Diet (MD) is a healthy dietary eating pattern and is characterized by an increased intake of plant-based foods, including fruits and vegetables, whole grains, legumes, nuts and seeds, and olive oil [7]. It is also characterized by a moderate intake of fatty fish, dairy, and poultry, and a low intake of red meats [7]. Research demonstrates that the MD is associated with a reduced risk of chronic diseases such as cardiovascular disease and Type 2 Diabetes. Recently, a literature review presented associations between the MD and mental health conditions such as depression and anxiety [8]. This review found that a few studies have shown a significant association with following a MD and improvement of depressive symptoms [8]. However, more studies are required to determine the exact effects the MD has on specific mental health disorders [8]. A 2023 systematic review conducted by Liu investigated the relationship between MD adherence and risk of depression [9]. A majority of studies included in the 2023 review found a significant association between MD adherence and depression risk [9]. But, further research should be conducted to determine the effectiveness of MD adherence on depression to determine treatment methods [9]. Additionally, more investigation on following a MD and its association with depression in specific populations, such as pregnant women, is needed due to the gaps in previous reviews.

To date, there have been no reviews conducted on the association between MD adherence and incidence of depression during pregnancy. Therefore, a review of recently published research considering the MD to ameliorate depression in pregnant women is imperative. The aim of this review is to determine if high adherence to the Mediterranean Diet or Mediterranean-like Diet (MD-like), compared to low or no adherence, is associated with a decreased incidence of depression or depressive symptoms during pregnancy.

## 2. Materials and Methods

This review was conducted by searching for articles on 27 February 2024, 22 May 2024, 11 June 2024, and 11 February 2025 using PubMed as the research database. PubMed, which indexes the biomedical and life sciences literature, was used to ensure only peer-reviewed papers were included and any other types of literature, such as dissertations that are commonly reviewed by peers inside the university, were excluded. The key search terms used were “Mediterranean Diet”, “Healthy Diet”, “Pregnancy”, and “Depression”. These terms were searched with the following search string: (“mediterranean diet” OR “healthy diet”) AND pregnancy* AND depression*. The full methods of the search and search syntax can be found in Appendix A. The searches were filtered to only include studies that were primary research articles and were published after 2019. The remaining studies went through a primary screening to ensure they were peer-reviewed articles. These articles went through a secondary screening based on the inclusion criteria listed in the following paragraph.

The inclusion criteria for this review included the following: studies conducted between 1 January 2019 and 11 February 2025 to ensure all studies were recent; studies that were primary research articles to ensure the authors conducted their own study on the topic; studies with the intervention diet of interest which is a Mediterranean Diet or a Mediterranean-like Diet which is a diet comprising a higher consumption of fruits, vegetables, plant proteins, dairy, whole grains, seafood, and a lower consumption of added sugars and saturated fat; studies that included participants that were pregnant and between the ages of 18 and 50 years old due to this being the population of interest; and studies where the outcomes of interest were reported which consisted of the incidence of prenatal depression or prenatal depressive symptoms. Studies needed to be published in the English language to be included.

Articles were first screened based on titles and abstracts for target interventions and outcome, then were screened further for all other inclusion criteria within the full-text version of the article. The variables extracted and reasoning for extraction are in Table 1. All abstracted data from each article appear in Table 2, Table 3 and Table 4. Table 2 provides the characteristics of the studies, Table 3 lists the statistical results, and Table 4 presents the strengths and limitations of each study.

## 3. Results

### 3.1. Search Results

**Figure 1:**
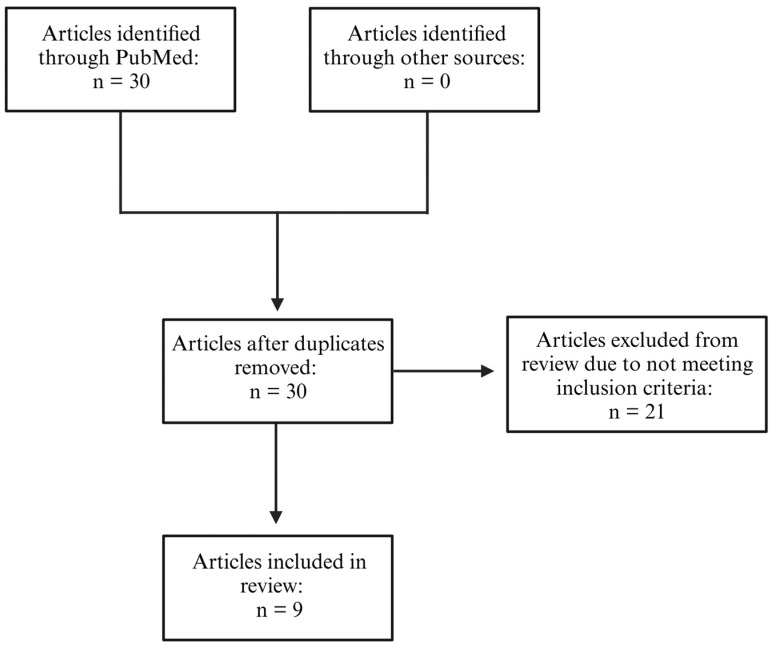
Flow diagram summarizing inclusion of articles for this literature review.

Figure 1 provides a flowchart of the search results included for this review. A total of 28 articles were identified, with 0 duplicates. After a full-text review of the articles, 21 studies were excluded due to not meeting the inclusion criteria for this review. Most of the excluded articles had a dietary intervention that did not meet the criteria for a MD or MD-like or the participants were not pregnant. This resulted in nine articles being included in this literature review.

### 3.2. Study Characteristics

Nine studies are included in this review. Each of the nine studies in the literature review are listed with summarizing information in Table 2 and Table 3. Importantly, the definition of the MD or MD-like for each study is provided in Table 2.

The American Academy of Nutrition and Dietetics Quality Checklist Tool was used to assess each study for bias [19]. Each study received a ‘+’ indicating relevance and validity. Of the nine studies, five were cross-sectional studies, three were longitudinal cohort studies, and one was a randomized controlled trial. In total, 10,042 pregnant mothers aged 18–40 years old were included in this review. The proportion of White participants ranged from 34.1 to 95.7% in the six studies that reported race of participants [10,11,12,14,15,17]. Two of the studies were conducted in Greece, four were in various locations in the United States, one was conducted in each of the following: Vietnam, Canada, and Spain. Different tools were used to measure depression and intervention diet, which may be related to location of the study (Table 2). Each study had several strengths and limitations, which are reviewed in Table 4.

### 3.3. Association Between MD or MD-like and Depression in Pregnant Women

There have been mixed results to interpret based on how the MD or MD-like is associated with depression or depressive symptoms in pregnant women. When comparing each study included in this review, significant associations between higher MD or MD-like adherence and a lower incidence of depression or depressive symptoms were found in six out of the nine studies [10,11,14,15,16,17]. The magnitude of the relationship found between the MD or MD-like and depression ranged from a lower likelihood of depression during pregnancy when following a MD-like to a more than two-fold lower frequency of perinatal depression with high MD adherence [10,11,12,13,14,15,16]. The magnitude of the relationship found between the MD or MD-like and depressive symptoms ranged from low MD compliance noted more often in depressed pregnant women to higher MD adherence associated with lower odds of moderate–severe depressive symptoms [10,17]. Table 5 provides a simplified summary of the outcomes reported from studies with significant associations between MD or MD-like adherence and depression or depressive symptoms. Three out of the nine studies found significant associations between specific food groups of a MD or MD-like and lower incidences of depression or depressive symptoms [11,12,13]. One out of the nine studies indicated that higher MD adherence had an association with lower incidence of anxiety and tended to decrease depression; but in this study, there was only a possible trend observed between higher mediterranean diet adherence and lower incidence of depression [18]. Thus, in this review, 67% of the included publications concluded that higher adherence to the MD or MD-like decreased the incidence of depression or depressive symptoms in pregnant women.

### 3.4. Association Between Specific Foods Groups and Depression in Pregnant Women

As stated above, three out of the nine studies found significant associations between specific food groups of a MD or MD-like and lower or higher incidences of depression or depressive symptoms [11,12,13]. Focusing on the outcome, two of these studies analyzed which specific foods increase the incidence of depression [11,12], and one of these studies focused on which specific food groups decrease the incidence of depression [13]. Conversely, focusing on the exposures, one study focused on higher consumption of specific foods and their association with depression or depressive symptoms [12], one study focused on lower consumption of specific foods and their association with depression or depressive symptoms [11], and one study focused on both higher and lower consumption of specific foods and their association with depression or depressive symptoms [13]. Based on these three studies, the only food group included in more than one study that had a significant association with depression was fruit [11,13]. One of the studies found that higher consumption of fruit was associated with a decreased incidence of depression in pregnant women [13], and another study found that lower consumption of fruit was associated with an increased incidence of depression in pregnant women [11]. Thus, the evidence suggests that consumption of fruit is associated with a decreased incidence of depression. Table 6 provides an overview of the specific food groups in each of the three studies associated with depression during pregnancy.

### 3.5. Association Between Race and Depression in Pregnant Women

In this review, four out of the nine studies drew conclusions about the impact of race on the associations between MD or MD-like and depression during pregnancy [11,12,14,15]. In one study where it was found that women with prenatal depression had almost twice the odds of a poor diet quality in comparison to women without prenatal depression, this relationship was reported to potentially be stronger in Hispanic women [11]. However, race did not moderate the relationship between depressive symptoms and diet quality in another [12]. Though all three inverse associations were significant, the negative association between MD adherence and depression score was greater among Hispanic women (β = −0.91) and Black/African American women (β = −0.44) compared with White women (β = −0.19) in the third study [14]. Finally, another study found associations between higher MD adherence and a lower risk of depression in White participants [15], but, in the same study, people of color were not observed to have fewer depressive symptoms with higher MD adherence [15]. It is important to note that this study [15] does not define the specific races included in the people of color variable. For five of the nine studies, the majority of the study participants were White [10,11,12,15,17], one study had more Black participants compared to White and Hispanic participants [14], and the remaining three studies did not report on race [13,16,18]. Two studies that reported results related to race and demonstrated that race may have an association with depression during pregnancy had a majority (54.5–85.0% of total sample) of White participants [11,15]. In summary, there were conflicting results for race with some finding the MD to be more protective against depression in Hispanic pregnant women.

### 3.6. Study Duration

In terms of study duration, five out of the nine studies were conducted for three to four years [10,12,13,15,18]. Two were conducted for a duration of two years [14,18], one was conducted for a duration of three months [16], and one used data from a span of 13 years [17]. However, five out of the nine studies only evaluated participants at one time during pregnancy, thus participants were not followed throughout the entire study period [12,14,15,16,17]. The remaining four studies measured diet or outcomes two different times during pregnancy [11,13,18] or the pregnant mothers were followed throughout pregnancy and for nine months post-partum [10]. Study duration was not associated with results. Even the shortest study duration (three months) evaluated women at one point during their pregnancy, and this study had a large sample size resulting in a significant inverse association between MD or MD-like adherence and depression/depressive symptoms during pregnancy [16]. Table 7 provides an overview of the pregnancy stage for participants in each study.

### 3.7. Sample Size

There was a wide range of sample sizes for the studies included in this review. Six out of the nine studies included over 500 pregnant women in the study [10,11,14,15,16,17]. Two studies included over 100 pregnant women [12,13], and one included less than 50 pregnant women and was a randomized controlled trial [18]. The six studies with larger sample sizes (n > 500) found inverse associations between MD or MD-like adherence and depression during pregnancy [10,11,14,15,16,17]. The studies that did not find associations between overall MD or MD-like adherence, but did find significant associations between specific food groups and depression or depressive symptoms during pregnancy were the two studies that had over 100 pregnant women but fewer than 500 [12,13]. Lastly, the single study that found there was a possible trend between higher MD adherence and lower incidence of depression during pregnancy was the study with fewer than 50 participants [18]. However, this study was a randomized controlled trial. Combined, the information regarding sample size and associations between MD/MD-like and depression indicates that a sample size of fewer than 500 may be insufficient to observe associations between MD adherence and depression. The studies included in this review with smaller sample sizes reported only non-significant associations or possible trends between MD or MD-like adherence and depression or depressive symptoms during pregnancy.

## 4. Discussion

Based on this review, recent evidence suggests that high adherence to the MD or MD-like may decrease the incidence of depression or depressive symptoms of pregnant women compared to low or no adherence. Although there were mixed results when comparing all nine of the studies, six of the nine studies did find a significant association between higher MD or MD-like adherence and lower incidence of depression or depressive symptoms [10,11,14,15,16,17]. Two studies did not find significant associations with the overall diet [12,13], and one study found a possible trend for a decreased incidence of depression with higher adherence to a MD/MD-like, but the significance of this result is unclear given that depression symptoms also decreased in the control group of that randomized controlled trial [18]. The magnitude of the relationship observed between the MD or MD-like and depression ranged from a lower likelihood of depression during pregnancy when following a MD-like to a more than two-fold lower likelihood of perinatal depression with high MD adherence [10,11,12,13,14,15,16]. Further, the magnitude of the relationship observed between the MD or MD-like on depressive symptoms ranged from less MD compliance being considerably more often noted in depressed pregnant women to higher MD adherence being associated with lower odds of moderate–severe depressive symptoms [10,17]. Although more than half of the studies reported a significant association between higher adherence to the MD or MD-like and a decrease in the incidence of depression or depressive symptoms in pregnant women, there were few studies published on this specific topic and even fewer studies that fit the inclusion criteria. Thus, more research including a more diverse population with a larger sample size (i.e., n >500) is needed in the future to further investigate this association between the MD or MD-like and depression or depressive symptoms in pregnant women. The following paragraphs will discuss different variables that may contribute to the associations reported in the studies included in this review. These variables could be further investigated in future research.

In terms of specific food groups that increased or decreased the incidence of depression or depressive symptoms in pregnant women, fruit was the only food group that was indicated in more than one of the nine studies [11,13]. Thus, it is imperative to investigate whether fruit impacts the incidence of depression alone, and the potential biological mechanisms that form the basis for this association. A systematic review conducted by Novi Arfirsta Dharmayani et al. in 2021 investigated associations between fruit and vegetable intake and depressive symptoms in young people and adults aged 15–45 years old and found that increased fruit consumption is associated with decreased risk of developing depression based on the cohort studies included [20]. However, mixed results were found when analyzing the included studies’ results for the association between vegetable consumption and depression [20]. The Dharmayani review discusses the potential mechanisms for fruit and vegetable consumption decreasing depression, but states that more research is needed to determine exact mechanisms [20]. Some studies have found an association with a decrease in depression and specific nutrients including magnesium, zinc, and antioxidants such as vitamins C, E, and folate which are commonly found in fruits and vegetables [20]. Folate has specifically been investigated related to its association with a decreased incidence of depression, and folate is found in many vegetables and citrus fruits [20]. Folate is important for the regeneration of components, such as tetrahydrobiopterin (BH4) and S-adenosylmethionine (SAMe), which are involved in producing neurotransmitters, including serotonin, dopamine, and epinephrine, needed for mood regulation [20]. The Dharmayani review and this present review closely align because both reviews found that fruit had a strong inverse association with depression. However, the Dharmayani review did not include studies with pregnant women. Sydney’s “Centre for Healthy Brain Ageing” discusses how certain components (including antioxidants, dietary fiber and vitamins) found in fruits and vegetables may have a beneficial influence on depression through many different mechanisms such as their role in inflammation, the gut microbiota, and oxidative stress [21]. Due to fruits and vegetables having varying types and amounts of nutrients, it is hypothesized that different types of fruit and vegetables may have distinct effects on depression [21]. Thus, consumption of fruit is associated with a decreased incidence of depression; however, the exact mechanisms which form the basis for this association require more research.

It is important to note that race was highlighted in the results in two of the nine studies, in which most of the participants were White [11,16]. Only one of the two studies found that White participants had a significant association between a decreased incidence of developing depression or depressive symptoms during pregnancy and a higher adherence to the MD while other races did not [16]. The other study includes in the results that this association between MD-like adherence and reduced depression may be stronger in the Hispanic population [11]. With that being said, five of the nine studies had a majority of White participants included [10,11,12,15,17]. This leads to the assumption that there may be a potential bias in the results of this review because White participants were the majority in more than half of the studies included. Race is an important factor that can affect maternal outcomes, such as depression, due to racism, lack of insurance coverage, limited availability of mental health professionals, and social factors including a lack of transportation or childcare [22]. Additionally, the MD dietary pattern has traditionally been eaten in the Mediterranean and has been adopted as a healthy diet in other countries such as the United States [23]. But adhering to the MD may not be feasible due to cultural and socioeconomic reasons [23], which can be associated with race. However, one of the five studies with a majority of White participants did not detect a significant association between MD or MD-like adherence and a decrease in depression or depressive symptoms [12], and there did not appear to be any differences in the other variables included in this study [12] that would make it unique from the other studies with a majority of White participants. Thus, determining if race may affect the observed inverse relationship between MD adherence and depression is something that should be further investigated.

There were different definitions for both the MD and MD-like. Six of the nine studies specially denoted that the MD was the intervention being studied [10,13,14,15,17,18], while three out of the nine studies denoted a healthy eating pattern as the intervention that was measured by the Healthy Eating Index (HEI)-2010, HEI-2015, or the Healthy Eating Score [11,12,16]. Of the six studies including the MD as the intervention diet, differences in the definition included different names for food groups, removal of certain food groups to moderately consume or limit, and some including monounsaturated fatty acid to saturated fatty acid ratios in the definition [10,13,14,15,17,18]. MD-like definitions differed between the three studies based on differing names of food groups and a lack of certain food groups [11,12,16]. A literature review conducted in 2015 by Davis, Bryan, Hodgson, and Murphy discusses the substantial evidence that the MD is beneficial for health-related outcomes including cognitive health while highlighting that there are inconsistencies in defining the diet in the literature [24]. This can make it difficult for researchers to determine if the true MD is associated with the outcome of interest when the definition can differ from study to study. A review conducted in 2011 by Bach-Faig et al. discusses the definition of the MD to help provide a universal representation of the MD food pattern in the Mediterranean area [25]. This 2011 review states that the MD pattern includes fruits, vegetables, and cereals to be consumed at each meal. Cereals are primarily whole grain. Low-fat dairy, nuts, and seeds are consumed daily as well [25]. Olive oil, which is rich in monounsaturated fatty acids, is the main fat source in meals [25]. Two or more servings of fish, white meat, and eggs are to be eaten weekly [25]. Two or less servings of red meat and one or less servings of processed meat are consumed weekly [25]. Three or less servings of potatoes are consumed weekly [25]. Lastly, sugar, candies, pastries, and sweetened beverages should be limited and only consumed in small amounts occasionally [25]. Overall, the MD diet is a plant-based food dietary pattern along with the consumption of healthier fats [26]. Based on the 2011 review’s MD definition, none of the studies in this present review provide such detail in the definition of the intervention diet. However, it is important to note that none of the studies included a food group to increase, moderately consume, or limit that was not aligned with the MD [10,11,12,13,14,15,16,17,18]. Instead, there were certain components of the diet missing in the definition or a lack of clarity in the definition itself. Additionally, there was no correlation between the definition of the MD or MD-like diet and outcome because two of the three studies with a MD-like diet found that adherence was associated with a decreased incidence of depression or depressive symptoms [11,16], and four out of the six studies with a MD diet found that adherence was associated with a decreased incidence of depression or depressive symptoms [10,14,15,17]. Future research should clearly define the intervention diet utilized to ensure the diet definition is consistent across all studies. Alternatively, future research could determine if any plant-based, healthy dietary pattern has the same effects as the MD/MD-like on depression or depressive symptoms during pregnancy.

No reviews to date include the same inclusion criteria focusing on the MD and incidence of depression during pregnancy. However, prior reviews that focus on comparing a healthy diet pattern to a traditional or unhealthy diet and how these affect depression in pregnant women have been published. A review conducted in 2020 by Boutté et al. aimed to examine associations of stress and/or depressive symptoms with diet quality during pregnancy [27]. The Boutté review discusses different food groups and how each related to good diet quality, including fruits, vegetables, grains, and dairy [27]. There was a total of 27 studies included in this review [27], of which none overlapped with the studies included in this present review. It was found that there was a higher incidence of depressive symptoms with a high unhealthy dietary pattern score and with a lower healthy dietary pattern score [27]. Thus, the 2020 review had similar findings to this present review in which a healthier eating pattern is associated with a lower incidence of depression during pregnancy. But again, the Boutté review does not discuss the MD or a diet that is Mediterranean-like, rather they included all studies that examined a healthy eating pattern and associations of such dietary patterns with depression during pregnancy. Another review conducted by Sparling, Henschke, Nesbitt, and Gabrysch in 2016 aimed to synthesize the recent literature on dietary intake and the risk of depression in women during the perinatal period [28]. Dietary intake is defined as adherence to certain diets, or food-derived intake of essential nutrients or supplementation [28]. This review found that there is limited evidence on the influence of dietary intake and perinatal depression; the results were inconclusive based on the studies included [28]. None of the studies included in the Sparling review overlapped with the studies included in this present review. The 2016 review along with the 2020 review both discuss the limited research on diet and the incidence of depression during pregnancy [27,28]. This present review also concludes that more research is needed on this topic. Thus, these reviews [27,28] agree with the conclusion of the present review: additional high-quality studies are necessary to better understand the association between diet and the incidence of depression during pregnancy.

Other reviews have been conducted on the association between the Mediterranean diet adherence and depression, but the inclusion criteria differed from this present review in terms of study population. A few to highlight include a review conducted by Zielińska, Łuszczki, Michońska, and Dereń in 2022 where the study aimed to analyze data on the dietary patterns and composition of the MD as a risk factor for depression in adolescents [29]. The 2022 study found that the MD pattern reduces the risk and symptoms of depression, while western dietary patterns increase the risk and severity of depression in adolescents [29]. Thus, the 2022 study and this present study found similar results in that the MD may have an association with a decrease in depression risk; however, the present review focuses on pregnant women whereas the 2022 review focuses on adolescents. Another review conducted by Gianfredi et al. in 2023 aimed to assess the available evidence on associations between many different dietary patterns and depression in adults older than 18 years [30]. This study found a weak strength of evidence on different dietary patterns overall, but there is convincing evidence to date on an inverse relationship between MD and depression [30]. Higher adherence to the Mediterranean Diet was significantly associated with a lower risk of depression [30]. This 2023 study looks at adults and the association between diet and depression and did not include pregnant individuals. Regardless of the difference in the inclusion criteria for study population in the 2022 and 2023 studies, similar results were observed in that adherence to a MD or MD-like diet may be associated with a decreased risk for depression. It is expected that the underlying mechanisms of the observed associations are similar. These two additional reviews also concluded that there is not a lot of research that has been conducted on this topic and more is required to further understanding of the Mediterranean Diet and depression association [29,30], and this present review notes there is even less research on this association in pregnant women. In the future, more high-quality studies are required to fully describe the association of the MD or MD-like and the decreased incidence of depression in different populations to further understand the significance of this association.

### 4.1. Strengths

One strength of this review is that the included studies were not all conducted in the Mediterranean, rather many were conducted in the United States where the MD is not the traditional diet pattern. This provides less bias to the adherence of the MD because not all participants in these studies would normally follow this diet due to their culture or traditions. Another strength of this review is that the sample sizes for over 50% of the studies were >500 participants, providing a large sample size [10,11,14,15,16,17]. Additionally, each study included a definition of the intervention diet, which made it easier to compare between studies and effectively evaluate if the diet aligned with the MD. Studies were evaluated for bias and rigor, and all studies met the criteria. Lastly, the studies included in this review were all recent publications from within the past five years.

### 4.2. Limitations

One limitation of this review is the potential for publication bias which may impact the results of this review. It is possible that studies were not published due to the authors not finding the results they were intending to find in terms of associations between the MD or MD-like and incidence of depression or depressive symptoms. Directionality of the studies is another limitation, as the majority of the included studies were cross-sectional. Thus, it cannot be determined whether MD adherence affects depression or if depression affects MD adherence in pregnant women. Additionally, there were certain variables to extract that were missing from several studies. Although all studies received a ‘+’ for quality research based on the Academy of Nutrition and Dietetics’ checklist, this lack of information means the full study design is ambiguous. Lastly, the included studies presented different epidemiological designs and used different methodologies/instruments to measure the outcomes studied. Thus, the conclusions made from this review should be assessed with caution.

## 5. Conclusions

This review provides evidence to suggest that high adherence to the MD or MD-like may decrease the incidence of depression or depressive symptoms in pregnant women. Six out of the nine studies did find a significant association between higher MD or MD-like and lower incidence of depression or depressive symptoms [10,11,14,15,16,17]. However, there were mixed results found when comparing all nine of the studies included in this review. Thus, more high-quality research needs to be conducted to further investigate if the MD or MD-like decreases the incidence of depression or depressive symptoms in pregnant women. This review only included nine primary research articles due to the limited number of studies conducted that met the inclusion criteria, and there have been no prior review articles written on this topic. Future studies must determine what components, if any, of the MD decrease the incidence of depression. Additionally, future research should investigate if the relationship between MD or MD-like adherence and decreased incidence of depression in pregnant women persists in study populations with a larger proportion of non-White participants, or participants of low socioeconomic status, since cultural and socioeconomic factors may result in limited adherence to this diet [23]. Lastly, it is imperative that future research includes a clear and unified definition of the MD because past studies have included either vague definitions or slightly different versions of the MD which makes it difficult to draw conclusions about specific aspects of the diet. With depression during pregnancy being a detrimental illness that can affect both the child and the mother negatively, it is imperative to continue investigating how a dietary pattern, such as the MD, can decrease the incidence of this disease during the perinatal period, a crucial time in the lives of both mothers and children.

## Figures and Tables

**Table 1:** Variables extracted and reasoning for extraction in this review.

Data Extracted	Reason for Extraction
Author/Publishing Date	To ensure the paper was published between 1 January 2019 and 11 February 2025.
Dates of Study	To ensure the reader is aware of the data collection date(s) since data may have been collected years prior to study publication.
Study Design	To ensure the studies were primary research articles.
Study Location	To ensure studies were not conducted in the same location, which could lead to potential bias.
Participants: Sample size, Pregnancy Status	To ensure the participant’s characteristics aligned with population of interest.
Mediterranean Diet Definition	To ensure the intervention diet aligned with the definition of a MD or MD-like.
Survey Instrument to Measure Outcomes	To ensure the instrument utilized was a validated tool to assess depression or depressive symptoms.
Outcomes: Depression and/or Depressive Symptoms	To ensure either one or both outcomes of interest were included in the results section of papers.
Strengths and Limitations	To describe strengths and limitations of each paper.

**Table 2:** Characteristics of the nine studies included in this review.

Author/Publish Date	Dates of Study	Study Design	Study Location	Participants: Sample Size, Pregnancy Status	MD/MD-like Definition	Survey Instrument to Measure Outcomes
Jacovides, 2024 [10]	May 2016–September 2020	Cross-Sectional	Greece	5314 pregnant women	High in fruits/vegetables (F/V), whole grains, nuts, seeds, beans, and olive oil, moderate in fish, poultry, dairy products and wine, and low in red meat and sweets. Applied the MedDietScore.	Beck Depression Inventory (BDI-II, during pregnancy), Edinburgh Postnatal Depression Scale (EPDS, post-partum)
Avalos, 2020 [11]	October 2011–April 2013	Cross-Sectional	Northern California, USA	1160 pregnant women	Increased F/V, whole grains, low-fat milk and milk products, seafood, lean meats and poultry, eggs, beans/peas, nuts and seeds with decreased intake of sodium, solid fats, added sugars, and refined grain. Applied the Healthy Eating Index (HEI)-2010 to data collected using a modified Block Food Frequency Questionnaire (FFQ)	Patient Health Questionnaire (PHQ-9)
Boutté, 2021 [12]	January 2015–January 2019	Cross-Sectional	Columbia, South Carolina, USA	228 pregnant women	Foods to increase: total and whole fruits, total vegetables, greens, beans, whole grains, dairy, total protein foods, seafood and plant proteins, and fatty acids. Foods to reduce: refined grains, sodium, added sugars, and saturated fats. Applied the Healthy Eating Index (HEI)-2015 to two 24-h recalls	10-item Edinburgh Prenatal/Postnatal Depression Scale (EPDS)
Flor Alemany, 2022 [13]	November 2015–April 2018	Longitudinal study	Granada, Spain	152 pregnant women	Consisting of olive oil, fiber, F/V, fish, cereals, meat, and alcohol. Applied the Mediterranean Food Pattern, an operational healthy diet score, to a Food Frequency Questionnaire (FFQ)	20-item Spanish version of the Center for Epidemiological Studies Depression Scale (CES-D)
Gonzalez-Nahm, 2022 [14]	2009–2011	Longitudinal cohort study	North Carolina, USA	929 pregnant women	Includes food to increase: F/V, fish, dairy, whole grains, legumes, nuts, and monounsaturated fats, (ratio of monounsaturated fatty acids to saturated fatty acids). Foods to decrease: meat. Applied the Mediterranean Diet Score to data collected by modified block food frequency questionnaire (FFQ).	CES-D
Vaghef-Mehrabani, 2024 [15]	2009–2012	Longitudinal cohort study	Calgary or Edmonton, Canada	1141 pregnant women	MD including high intake of whole grain cereals, legumes, F/V, nuts, herbs), moderate intake of seafood, dairy, and poultry, and limited amounts of red meat, sweets, and red wine. Calculated a modified MED adherence score using data collected with a food frequency questionnaire (FFQ) based on the National Cancer Institute’s Diet History Questionnaire for Canadians.	EPDS
Luong, 2021 [16]	14 February–31 May 2020	Cross-Sectional study	Vietnam	518 pregnant women	Higher consumption of F/V, whole grains, low-fat dairy and fish based on a 5-item Healthy Eating Score.	PHQ-9
Oddo, 2023 [17]	2005–2018	Cross-Sectional, National Health and Nutrition Examination Survey (NHANES)	USA	540 pregnant women	Higher consumption of F/V, whole grains, nuts, fish, and a higher monounsaturated fatty acids/saturated fatty acids ratio. Lower consumption of red and processed meats. A Mediterranean Diet Score, modified for use with 24 h diet recall, was calculated using data from a 1-day 24 h diet recall.	PHQ-9
Papandreou, 2023 [18]	May 2019–May 2022	Randomized Control Trial	Athens, Greece	40 pregnant women	MD entails a high intake of F/V, whole grains cereals, legumes, fish, nuts, and olive oil as the main source of fat. Applied the MedDietScore to data collected by a modified, semi-quantitative, food frequency questionnaire (FFQ).	The Hospital Anxiety and Depression Scale (HADS)

Abbreviations: BDI-II, Beck Depression Inventory; CES-D, Center for Epidemiological Studies Depression Scale; EPDS, Edinburgh Postnatal Depression Scale; FFQ, food frequency questionnaire; F/V, fruits and vegetables; HADS, Hospital Anxiety and Depression Scale; HEI, Healthy Eating Index; MD, Mediterranean diet; PHQ-9, Patient Health Questionnaire; USA, United States of America.

**Table 3:** Summary of statistical results for all nine studies included in this review.

Author/Publish Date	Outcome 1: Depression	Outcome 2: Depressive Symptoms
Jacovides, 2024 [10]	Women reporting enhanced MD compliance had a more than two-fold lower frequency of perinatal depression during their pregnancy. (Moderate + High/Very Low + Low) MD adherence: Hazard Ratio (HR) (95% CI) 2.18 (1.97–2.40) (*p* = 0.0011).	Lower levels of MD compliance were considerably more often noted in depressed pregnant women compared to non-depressed ones. NO/YES perinatal depressive symptoms proportion w/MD adherence: Very low: 739/591; Low: 770/548; Moderate: 940/388; High: 1000/338 (*p* < 0.0001).
Avalos, 2020 [11]	Overall, women with prenatal depression had nearly twice the odds of poor diet quality (crude odds ratio: 1.89; 95% Confidence Interval (CI): 1.32, 2.69) compared to women without. The relationship may be stronger in Hispanic women. Women with a higher/lower consumption of specific foods and prenatal depression was also significant.	N/A
Boutté, 2021 [12]	N/A	Overall, depressive symptoms were not significantly related to Healthy Eating Index total scores (*p* = 0.36). However, higher levels of depressive symptoms were associated with intake of specific food groups (dairy, refined grains, and saturated fats).
Flor Alemany, 2022 [13]	There was a borderline non- significant association between MD adherence and depression (*p* = 0.066). The cross-sectional analysis showed a higher/lower intake of fruits was associated with lower depression (*p* = 0.036).	N/A
Gonzalez-Nahm, 2022 [14]	A 1-point increase in MD score was associated with negative 0.45-point difference in depression score during the first trimester. Depression score (CES-D): Beta (β) (95% CI) − 0.45 (−0.90, −0.18) (*p* = 0.017).	N/A
Vaghef-Mehrabani, 2024 [15]	White participants in Tertile 3 (highest adherence) had a 44% decreased risk of late pregnancy depression compared to those in Tertile 1 (less adherence). No significant association for people of color/pooled samples. Tertile 3 compared to Tertile 1 in White sample: Odds Ratio (OR), 0.56 (0.33, 0.95) (*p* < 0.05).	N/A
Luong, 2021 [16]	Pregnant women with higher scores of Healthy Eating Score and Health Literacy Score had lower likelihood of depression. OR, 0.84; 95% CI, 0.78, 0.91; (*p* < 0.001); and OR, 0.96; 95% CI, 0.91, 0.99; (*p* = 0.044).	N/A
Oddo, 2023 [17]	N/A	Higher (compared with lower) adherence to a MD was associated with lower odds of having moderate to severe depressive symptoms among pregnant women. Relationship between MD adherence and moderate to severe depressive symptoms; OR (95% CI): 0.31 (0.10, 0.98).
Papandreou, 2023 [18]	Depression levels tended to be reduced in the CDSS MD group (*p* = 0.054).	N/A

Abbreviations: CDSS, clinical decision support system; CI, confidence interval; HR, hazard ratio; MD, Mediterranean diet; OR, odds ratio; N/A, not applicable.

**Table 4:** Strengths and limitations of the nine studies included in this review.

Author/Publish Date	Strengths	Limitations
Jacovides, 2024 [10]	- Large sample size - Well-defined intervention diet	- Conducted in the Mediterranean
Avalos, 2020 [11]	- Large sample size - Well-defined intervention diet	- Study was limited to English-speaking women. The associations observed among the Hispanic women may not be representative of Hispanic women who do not speak English
Boutté, 2021 [12]	- Well-defined intervention diet	- Smaller sample size
Flor Alemany, 2022 [13]	- Conducted both longitudinal and cross-sectional analyses	- Conducted in Mediterranean - Small sample size
Gonzalez-Nahm, 2022 [14]	- Large sample size	- Duration of study not included in study
Vaghef-Mehrabani, 2024 [15]	- Large sample size - Well-defined intervention diet	- Study was limited to women who could read and speak English
Luong, 2021 [16]	- Large sample size	- Intervention diet not well defined
Oddo, 2023 [17]	- Large sample size - Well defined intervention diet	- Analysis of secondary data
Papandreou, 2023 [18]	- Randomized Control Trial	- Small sample size - Conducted in the Mediterranean region

**Table 5:** Simplified outcomes reported from studies with significant associations between MD or MD-like adherence and depression or depressive symptoms.

Author/Publish Date	Outcomes 1: Depression	Outcome 2: Depressive Symptoms
Jacovides, 2024 [10]	Enhanced MD compliance led to a two-fold lower frequency of perinatal depression during pregnancy.	Low MD compliance was considerably more often noted in depressed pregnant women compared to non-depressed women.
Avalos, 2020 [11]	Overall, women with prenatal depression had nearly twice the odds of poor diet quality compared to women without depression.	N/A
Gonzalez-Nahm, 2022 [14]	An increase in MD score was associated with a decreased depression score.	N/A
Vaghef-Mehrabani, 2024 [15]	White participants in Tertile 3 (highest adherence) had a 44% decreased risk of late pregnancy depression compared to those in Tertile 1 (less adherence).	N/A
Luong, 2021 [16]	Pregnant women with higher Healthy Eating Scores and Health Literacy scores had a lower likelihood of depression.	N/A
Oddo, 2023 [17]	N/A	Higher adherence to a MD was associated with lower odds of having moderate to severe depressive symptoms.

Abbreviations: N/A, not applicable.

**Table 6:** Single food groups associated with depression during pregnancy.

Author/Publish Date	Specific Foods Associated with Depression During Pregnancy	Specific Foods Associated with Depressive Symptoms During Pregnancy
Avalos, 2020 [11]	Reduced consumption of greens and beans, total fruit, and whole fruit was positively associated with the incidence of depression in pregnant women. Consumption of solid fats, alcohol, and added sugars was positively associated with depression.	N/A
Boutté, 2021 [12]	N/A	Higher levels of depressive symptoms during pregnancy were associated with greater consumption of - Dairy [β (Standard Error (SE)) 0.06 (0.02); *p* < 0.001] - Refined grains [β (SE) 0.11 (0.04); *p* < 0.01] - Saturated fats β (SE) 0.44 (0.17); *p* = 0.01]
Oddo, 2023 [17]	The cross-sectional associations showed a higher intake of whole grain cereals, fruits, vegetables, fish, and nuts, and a lower intake of red meat and subproducts and sweets were associated with lower depression (*p* < 0.05). The longitudinal associations showed a higher intake of fruits, olive oil, and nuts together with a lower intake of red meat and subproducts was associated with lower depression (*p* < 0.05).	N/A

Abbreviations: N/A, not applicable.

**Table 7:** Timing during pregnancy for participants in each study.

Author, Publish Date	Timing During Pregnancy
Jacovides, 2024 [10]	Enrollment: 3rd–6th month of gestation. Women were followed until 9 months post-partum.
Avalos, 2020 [11]	Outcomes were reported during the 1st and 24th week of pregnancy. The median gestational age when diet was evaluated was 25 weeks. More than 75% of the women completed the diet evaluation at 16 weeks gestation or later.
Boutté, 2021 [12]	Women were ≤16 weeks gestation at baseline. Diet and outcomes were reported at baseline.
Flor Alemany, 2022 [13]	Cross-sectional (16th gestational week) and longitudinal associations (34th gestational week) between MD and mental health were studied.
Gonzalez-Nahm, 2022 [14]	Women’s periconceptional diet was measured at enrollment. Outcomes were evaluated based on the 2 weeks prior to evaluation.
Vaghef-Mehrabani, 2024 [15]	Women were recruited before 27th week gestation, and outcomes were evaluated in 3rd trimester.
Luong, 2021 [16]	Dietary intervention and outcome measures were assessed at any time during pregnancy.
Oddo, 2023 [17]	Dietary intervention and outcome measures were assessed at any time during pregnancy
Papandreou, 2023 [18]	Dietary intervention and outcomes were assessed before and at the end of the intervention during first trimester.

## Data Availability

No new data were created or analyzed in this study. All relevant data are included in the manuscript.

## Abbreviations

The following abbreviations are used in this manuscript:

MD	Mediterranean Diet
MD-like	Mediterranean-like Diet

## Appendix A

**Databases Searched:**

**A. PubMed:**

Search Date: 27 February 2024

Date Range: 2019–2024

**Search Terms:**

**PubMed:** (“Mediterranean diet”[All Fields] OR “healthy diet”[All Fields]) AND “pregnancy*”[All Fields] AND “depression*”[All Fields]

Search Date: 22 May 2024

Date Range: 2019–2024

**Search Terms:**

**PubMed:** (“Mediterranean diet”[All Fields] OR “healthy diet”[All Fields]) AND “pregnancy*”[All Fields] AND “depression*”[All Fields]

Search Date: 11 June 2024

Date Range: 2019–2024

**Search Terms:**

**PubMed:** (“Mediterranean diet”[All Fields] OR “healthy diet”[All Fields]) AND “pregnancy*”[All Fields] AND “depression*”[All Fields]

Search Date: 11 February 2025

Date Range: 2019–2025

**Search Terms:**

**PubMed:** (“Mediterranean diet”[All Fields] OR “healthy diet”[All Fields]) AND “pregnancy*”[All Fields] AND “depression*”[All Fields]
